# Revision of *Chaetocnema semicoerulea* species-group (Coleoptera, Chrysomelidae, Galerucinae, Alticini) in China, with descriptions of three new species

**DOI:** 10.3897/zookeys.463.8147

**Published:** 2014-12-12

**Authors:** Yongying Ruan, Alexander S. Konstantinov, Siqin Ge, Xingke Yang

**Affiliations:** 1 Key Laboratory of Zoological Systematics and Evolution, Institute of Zoology, Chinese Academy of Sciences, Beijing 100101, China; 2 Systematic Entomology Laboratory, USDA, ARS, Washington DC, USA; 3 University of Chinese Academy of Sciences, Beijing, 100039, China

**Keywords:** Coleoptera, species group, new species, China, flea beetles

## Abstract

Chinese species of *Chaetocnema semicoerulea* group are revised and three new species described as new: *Chaetocnema salixis* **sp. n.**, *Chaetocnema yulongensis* **sp. n.** and *Chaetocnema deqinensis* **sp. n.**. A key to all five species of this group occurring in China and the illustrations of habitus and genitalia are provided. A map of species distribution is given.

## Introduction

There are more than 400 species of *Chaetocnema* known in the world (Konstantinov et al. 2011). Approximately 43 species are known to occur in China; however, Chinese *Chaetocnema* species still remained mostly unknown and need to be collected, properly documented and ultimately revised. The Chinese *Chaetocnema picipes* species-group has been studied previously (Ruan et al. 2014), and this paper is a second contribution to the revision of *Chaetocnema* in China.

*Chaetocnema semicoerulea* species-group is similar to *Chaetocnema picipes* species-group (Ruan et al. 2014). They share a number of character states, such as: vertex sparsely and unevenly covered with punctures near each eye; frontal ridge narrow and convex; base of pronotum with two short longitudinal impressions; deep row of large punctures at base of pronotum present on sides, lacking in middle. *Chaetocnema semicoerulea* species-group can be differentiated from *picipes* species-group by the following character states: apical part of median lobe in ventral view with polygonal line on lateral sides; apex of median lobe in lateral view sinuated.

Species of the *semicoerulea* species-group usually feed on *Salix* or *Rubus*.

## Material and methods

Photographic technique, morphological terminology and anatomy method used in Ruan et al. (2014) are followed.

Distributional records of species are arranged from north to south. Province names in “Material” paragraphs are in bold.

**Abbreviations of collections:** BMNH, The Natural History Museum, London, United Kingdom; IZCAS, Institute of Zoology, Chinese Academy of Sciences, Beijing, China; HMB, Switzerland, Basel, Natural History Museum; SYSU, Sun Yat-Sen University, Gauangzhou, China; USNM, National Museum of Natural History, Washington DC, USA; ZMHB, Museum für Naturkunde der Humboldt-Universität, Berlin, Germany.

## Taxonomy

### *Chaetocnema semicoerulea* species-group

**Diagnosis**

Body length usually 1.60–3.00 mm. Apex of median lobe narrowing with polygonal line on sides, apical denticle weak or absent; median lobe in lateral view sinuated on apex; spermathecal receptacle pear-shaped, basal part of spermathecal duct straight; frontal ridge narrow and convex; vertex sparsely and unevenly covered with punctures near eyes; all rows of punctures on elytra regular.

In history, many authors erroneously used Tlanoma Motschulsky as a subgenus name in the genus *Chaetocnema*. This mistake was fully discussed by Konstantinov et al. (2011). They pointed out that *Chaetocnema concinna* Marsham is the correct type of genus *Chaetocnema* designated by Westwood 1838, instead of *Chaetocnema hortensis* (Geofrroy) desinated by Maulik (1926) which was erroneously used by many authors in history and recently; the valid name for subgenus should be *Chaetocnema* (s. str.) (subgeneric type *Chaetocnema concinna*) and *Udorpes* (subgeneric type *Chaetocnema splendens*); *Tlanoma* Motschulsky fact is a subjective junior synonym of *Chaetocnema* in the strict sense. We agree with their proposal to use *Chaetocnema* and *Udorpes* as two valid subgeneric names in the genus *Chaetocnema*. Based on the narrow and raised frontal ridge and sparse punctures near each eye, species of the *semicoerulea* group can be classified in the subgenus *Chaetocnema* (s. str.) Marsham. Although the same paper suggested not to use any subgeneric classification until rigorous phylogenetic analysis has been conducted in this genus, in this study the species group is used for practical reasons.

### Key to species of *Chaetocnema semicoerulea* species-group

**Table:** 

1	Male body length exceeds 2.00 mm; ratio of width of frontal ridge (excluding margin) to width of antennal socket (excluding margin) exceeds 1.20.	**2**
–	Male body length less than 1.90 mm; ratio of width of frontal ridge (excluding margin) to width of antennal socket (excluding margin) less than 1.10	**4**
2	Head with 15–20 punctures on vertex near each eye	***Chaetocnema semicoerulea* (Koch)**
–	Head with 7–10 punctures on vertex near each eye	**3**
3	Frontolateral angle of pronotum round or blunt; longitudinal groove and minute transverse wrinkles on ventral side of median lobe present	***Chaetocnema transbaicalica* Heikertinger**
–	Frontolateral angle of pronotum strongly protruding, acute laterally; longitudinal groove and minute transverse wrinkles on ventral side of median lobe absent	***Chaetocnema salixis* sp. n.**
4	Body copperish; apex of median lobe narrowly rounded, lateral view of median lobe evenly curved	***Chaetocnema yulongensis* sp. n.**
–	Body bronzish; apex of median lobe broadly rounded, maximum curvature of median lobe from lateral view situated apically	***Chaetocnema deqinensis* sp. n.**

#### Chaetocnema semicoerulea

Taxon classificationAnimaliaColeopteraChrysomelidae

(Koch)

Figure 1

Chaetocnema semicoerulea Koch, 1803: 40 (type locality: Germany, Rheinland, “Kusel und Meisenheim”; type missing *teste* Doguet, 1994); as *Haltica*.
Chaetocnema saltitans Stephens, 1831: 327 (type locality: “Suffolk”; type depository: unknown); Weise 1886: 760 (synonymized).
Chaetocnema meridionalis Allard, 1859: cv (type locality: “France méridionale”; type depository: unknown); as *Plectroscelis*; Heikertinger 1951: 211 (synonymized).
Chaetocnema saliceti Weise, 1886: 758 (as variety of *semicoerulea*; type locality: not given; type depository: ZMHB); Heikertinger 1951: 211 (synonymized).
Chaetocnema femoralis Weise, 1886: 758 (as variety of *semicoerulea*; type locality: not given; type depository: ZMHB); Heikertinger 1951: 211 (synonymized).

##### Distribution.

China (Heilongjiang); Russia [Siberia (Heikertinger 1951), Far East (Bukejs 2008)]; Middle Asia (Heikertinger 1951); Europe (Konstantinov et al. 2011).

##### Host plants.

*Salix alba*, *Salix purpurea*, *Salix triandra*, *Salix incana*, *Salix viminalis*, *Calamagrostis* sp., *Phalaris* sp. (Heikertinger, 1925); *Salix alba*, *Salix purpurea*, *Salix triandra*, *Salix viminalis*, *Salix elaeagonos* (Doguet, 1994); *Salix alba*, *Salix purpurea* (Fogato & Leonardi, 1980).

##### Description.

Body length: 2.05–3.00 mm, without head: 2.08–2.70 mm; body width: 1.24–1.67 mm. Ratio of elytron length at suture to maximum width: 2.26–2.46. Ratio of pronotum width at base to length at middle: 1.61–1.65. Ratio of length of elytron at suture to length of pronotum at middle: 3.13–3.16. Ratio of width of both elytra at base to width of pronotum at base: 1.15–1.16.

Elytron bronzish, blueish or copperish. Pronotum bronzish, greenish or copperish. Antennomere 1–2 completely yellow. Antennomeres 3–4 completely yellow or partly brown. The remaining antennomeres brown. Tibia and tarsi yellow. Pro- and meso-femur dark yellow. Metafemur brown. Tarsi dark yellow.

Head hypognathous. Frontal ridge between antennal sockets narrow and convex. Frontolateral sulcus present. Orbital sulcus deep. Suprafrontal sulcus relatively deep, well-defined, retuse. Ratio of width of frontal ridge (excluding margin) to width of antennal socket (excluding margin): 1.35–1.45. Vertex flat, situated on same level as orbit. Surface of vertex covered with 15–20 punctures next to each eye.

Base of pronotum with two longitudinal impressions. Deep row of large punctures at base of pronotum present on sides, lacking in middle. Pronotal base evenly convex. Sides of pronotum slightly convex with maximum width near base. Anterolateral prothoracic callosity protruding laterally forming a round angle. Posterolateral prothoracic callosity poorly developed. Diameter of pronotal punctures 2–4 times smaller than distance between them.

Elytra with convex sides. All rows of punctures of elytra regular. Elytral humeral callus well-developed. Interspace between stria of punctures on the elytra smooth. Numbers of lines of minute punctures on each interspace: 2–3.

First male protarsomere length to width ratio: 2.20–2.25. First and second male protarsomeres length to length ratio: 1.13–1.19. First and second male protarsomeres width to width ratio: 1.61–1.67. Length of metatibia to distance between denticle and metatibial apex: 2.37–2.45. Large lateral denticle on metatibia sharp. Metatibial serration proximal to large lateral denticle present, sharp. First male metatarsomere length to width ratio: 2.97–3.06. First male protarsomere maximum width to width at base ratio: 2.12–2.19. First and second male metatarsomeres length to length ratio, 1.71–1.78. First and second male metatarsomeres width to width ratio: 1.00–1.06. Third and fourth male metatarsomeres length to length ratio: 1.54–1.59.

Apical part of median lobe narrower than middle. Apical part in ventral view narrowing gradually with polygonal line on sides. Ventral longitudinal groove poorly developed, shallow or absent. Apical and basal parts of longitudinal groove usually subequal in width, wider than middle. Apical denticle in ventral view absent. Minute transverse wrinkles on ventral side present. Median lobe in lateral view sinusoidal near apex with maximal curvature situated medially.

Spermathecal receptacle pear-shaped. Spermathecal pump much shorter than receptacle. Apex of spermathecal pump cylindrical. Spermathecal receptacle piriform. Spermathecal pump attached to middle of receptacle top. Maximum width of receptacle situated at about middle. Basal part of receptacle wider than apical. Posterior sclerotization of tignum spoon shaped, wider than midsection. Midsection of tignum curved. Sides of midpart of vaginal palpus narrowed. Anterior end of anterior sclerotization narrowly rounded. Length of posterior sclerotization greater than width. Width of anterior sclerotization greater than width of posterior sclerotization.

**Figure 1.**
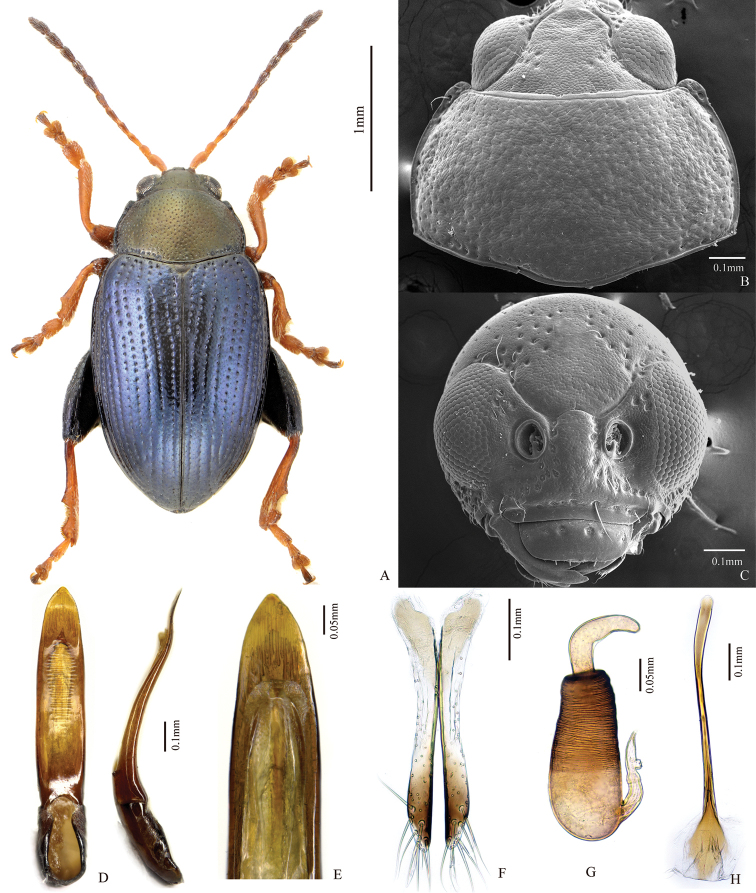
*Chaetocnema semicoerulea*. **A** Male habitus **B** Prothorax, dorsal view **C** Head, frontal view **D** Adeagus, ventral and lateral view **E** Apical part of Adeagus, dorsal view **F** Vaginal palpus **G** Spermatheca **H** Tignum.

##### Material.

1♂, China, Wuyiling, Yichun, **Heilongjiang**, 31.VIII.1970 (IZCAS).

##### Remarks.

*Chaetocnema semicoerulea* from the Palearctic Region has been recently revised by Konstantinov et al. (2011). This species is recorded in China for the first time. Only found one male specimen was found in China with all the characters in accordance with the European specimens that are available for study except the reduced body size (2.05 mm in body length).

#### Chaetocnema transbaicalica

Taxon classificationAnimaliaColeopteraChrysomelidae

Heikertinger

Figure 2

Chaetocnema transbaicalica Heikertinger, 1951: 173 (as subspecies of *semicoerulea*; type locality: Russia, Ulan-Ude, “Werchne Udinsk”; type depository: NHMB; lectotype designated by Bechyné 1956: 583); Konstantinov et al. 2011: 333 (elevated to species).

##### Distribution.

China (Heilongjiang); Mongolia; Russia (Southern Siberia, Far East) (Heikertinger 1951, Konstantinov et al. 2011).

##### Host plants.

unknown.

##### Description.

Body length: 2.05–2.25 mm, excluding head: 1.94–2.05 mm; Body width 1.18–1.24 mm. Ratio of elytron length at suture to maximum width: 2.59–2.68. Ratio of pronotum width at base to length at middle: 1.70–1.86. Ratio of length of elytron at suture to length of pronotum at middle: 3.28–3.38. Ratio of width of both elytra at base to width of pronotum at base: 1.07–1.19. Ratio of maximum width of both elytra to maximum width of pronotum: 1.40–1.44.

Elytron and pronotum bronzish. Antennomere 1 completely yellow or partly dark brown. Antennomeres 2–3 completely yellow. Antennomere 4 completely yellow or partly brown. Antennomere 5 partly brown. The remaining antennomeres brown. Tibia yellow or partly brown. Pro- and mesofemur partly brown. Metafemur brown. Tarsi yellow.

Head hypognathous. Frontal ridge between antennal sockets narrow and convex. Frontolateral sulcus present. Suprafrontal sulcus shallow and faint, retuse. Ratio of width of frontal ridge (excluding margin) to width of antennal socket (excluding margin): 1.30–1.40. Surface of vertex with 8–10 punctures near each eye.

Base of pronotum with two short longitudinal impressions. Deep row of large punctures at base of pronotum present on sides, lacking in middle. Pronotal base evenly convex. Sides of pronotum slightly convex with maximum width near base. Anterolateral prothoracic callosity protruding laterally. Posterolateral prothoracic callosity poorly developed. Diameter of pronotal punctures 2–4 times smaller than distance between them.

Elytra with convex sides. All rows of punctures on elytra regular, scutellar row single. Elytral humeral callus well-developed. First male protarsomere length to width ratio: 1.48–1.51. First male protarsomere maximum width to width at base ratio: 1.98–2.04. First and second male protarsomere length to length ratio: 1.79–1.83. First and second male protarsomeres width to width ratio: 1.06–1.09. Length of metatibia to distance between denticle and metatibial apex: 2.27–2.32. Large lateral denticle on metatibia sharp. Metatibial serration proximal to large lateral denticle present, sharp. First male metatarsomere length to width ratio: 2.96–3.05. First and second male metatarsomeres length to length ratio: 1.30–1.70. First and second male metatarsomeres width to width ratio: 0.85–0.91. Third and fourth male metatarsomeres length to length ratio, 1.63–1.69.

Apical part of median lobe in ventral view narrowing gradually with polygonal line on sides. Apical denticle in ventral view absent. Minute transverse wrinkles on ventral side present. Ventral longitudinal groove poorly developed in apical and basal part, shallow, with obtuse margins; obscure or absent in middle. Apical part of longitudinal groove wider than basal; middle part narrower than basal and apical. Median lobe in lateral view sinusoidal near apex with maximal curvature situated medially.

Spermathecal pump much shorter than receptacle. Apex of spermathecal pump cylindrical. Spermathecal receptacle pear-shaped. Spermathecal pump attached to middle of receptacle top. Maximum width of receptacle situated at about middle. Basal part of receptacle about as wide as apical. Posterior sclerotization of tignum spatulate, wider than midsection. Apex of vaginal palpus subdeltoid, with sides abruptly tapering. Sides of midpart of vaginal palpus (before apex) narrowing from base, slightly widening towards apex. Anterior sclerotization of vaginal palpus as wide posteriorly as anteriorly before apex; sharply curved at apex. Anterior end of anterior sclerotization broadly rounded or acute. Length of posterior sclerotization greater than width. Width of posterior sclerotization great or greater than width of anterior sclerotization.

**Figure 2.**
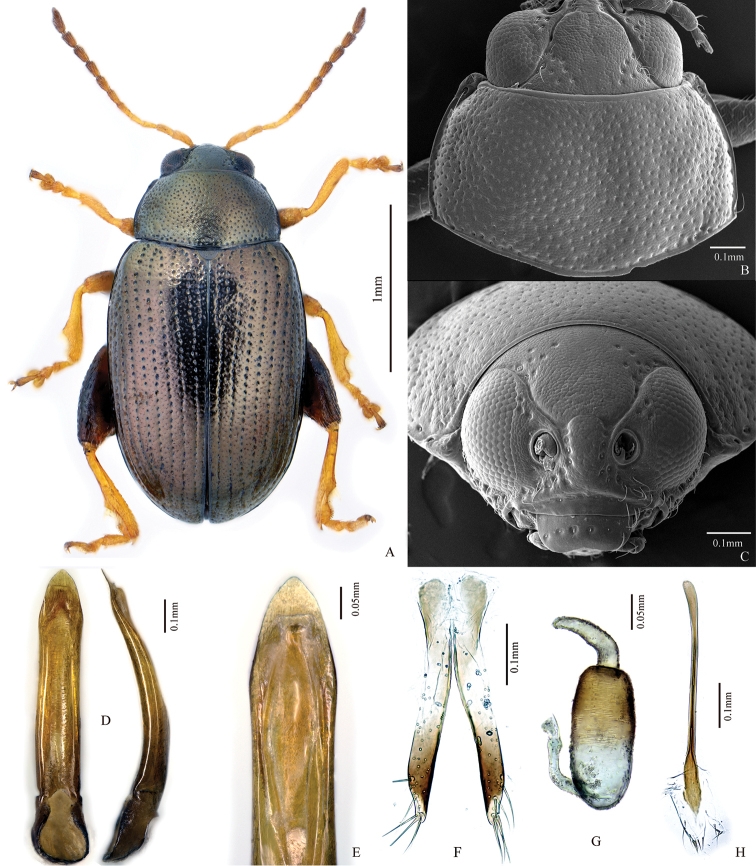
*Chaetocnema transbaicalica*. **A** Male habitus **B** Prothorax, dorsal view **C** Head, frontal view **D** Adeagus, ventral and lateral view **E** Apical part of Adeagus, dorsal view **F** Vaginal palpus **G** Spermatheca **H** Tignum.

##### Type material.

*Chaetocnema transbaicalica*: Lectotype: 1♂, 1) Werchne-Udinsk, Trabaikal. Mandl, 2) nicht, semicoer. Aedeagus, 1. Tarsingl., 3) Chaetocn., det. Heiktgr., semicoerul. transbaicalica m. Type, 4) Chaetocn. semicoer. transbaicalica m. Typus, 5) 1953, Coll. Heikertinger, 6) lectotype, J. Bechyné det., 1956 (NHMB);

Paralectotype: 1, 1) Sutschanski-Rudnik, Ussuri Juli, 2) semicoerulea transbaicalica m. det. Heiktgr., 3) Cotypus, 4) 1953, Coll. Heikertinger (NHMB); 1, 1) Werchne-Udinsk, Trabaikal. Mandl, 2) Punktiernug der Fld.anders!, 3) Chaetocn. semicoerul. Transbaicalica m. Type, det. Heiktgr., 4) Chaetocn. semicoer. transbaicalica m. Typus, 5) 1953, Coll. Heikertinger (NHMB).

##### Material.

2♂, China, Errenban, Mishan, **Heilongjiang**, 10.VIII.1970 (IZCAS); 1♀, Dabaishu, Daxinganling, **Heilongjiang**, 19.VIII.1970. 1♂1♀, Mongolia, 27.VI.1924 (IZCAS).

##### Remarks.

*Chaetocnema transbaicalica* from the Palearctic Region has been recently revised by Konstantinov et al. (2011). This species is previously recorded in Russia and Mongolia. This species is recorded in China for the first time.

#### Chaetocnema salixis

Taxon classificationAnimaliaColeopteraChrysomelidae

Ruan, Konstantinov & Yang sp. n.

http://zoobank.org/BA6C51B8-E65F-417B-B7B4-955FD7C9A53C

Figure 3

##### Etymology.

This species is named after the host plant.

##### Host plants.

*Salix* sp.

##### Distribution.

China: Shannxi, Gansu, Sichuang.

##### Diagnosis.

*Chaetocnema salixis* resembles *Chaetocnema transbaicalica*. It can be differentiated from the latter by the following characters: frontolateral angle of pronotum long and sharp laterally; both longitudinal groove and transverse wrinkles on ventral surface of median lobe absent; metatibial serration proximal to large lateral denticle absent or obscure.

##### Description.

Body length: 2.10–2.50 mm, without head: 1.9–2.30 mm. Female body length: 2.30–2.50 mm, without head: 2.10–2.30 mm. Length of antenna to length of body: 0.60. Body width: 1.13–1.17 mm. Elytron length (along suture) to width (maximum): 2.70–2.75. Pronotum width (at base) to length: 1.74–1.79. Length of elytron to length of pronotum (along middle): 3.47–3.49. Width of elytra at base (in middle of humeral calli) to width of pronotum at base: 1.16–1.19. Maximum width of elytra to maximum width of pronotum: 1.44–1.47.

Color of elytron differs from color of pronotum. Elytra black, without metallic lustre. Pronotum and head bronzish. Antennomere 1 partly dark brown. Antennomeres 2–3 partly brown or completely yellow. Antennomere 4 brown. The remaining antennomeres black. Tibia and tarsi partly brown. Femora brown.

Head hypognathous. Frontal ridge between antennal sockets narrow and convex. Frontolateral sulcus present. Suprafrontal sulcus deep laterally, absent in middle. Orbital sulcus deep. Ratio of width of frontal ridge (excluding margin) to width of antennal socket (excluding margin): 1.20–1.25. Width of frontolateral sulcus to width of antennal socket: 0.29–0.38. Width of orbital sulcus to width of frontolateral sulcus: 0.72. Numbers of punctures on vertex next to each eye: 7–9. Numbers of punctures on orbit: 2. Numbers of hairs along frontolateral sulcus: 9–11. Numbers of hairs on front (triangle area surrounded by frontolateral sulcus and clypeus): 0. Numbers of hairs on clypeus: 8. Numbers of hairs on labrum: 6. Anterior margin of labrum slightly concave in middle.

**Figure 3.**
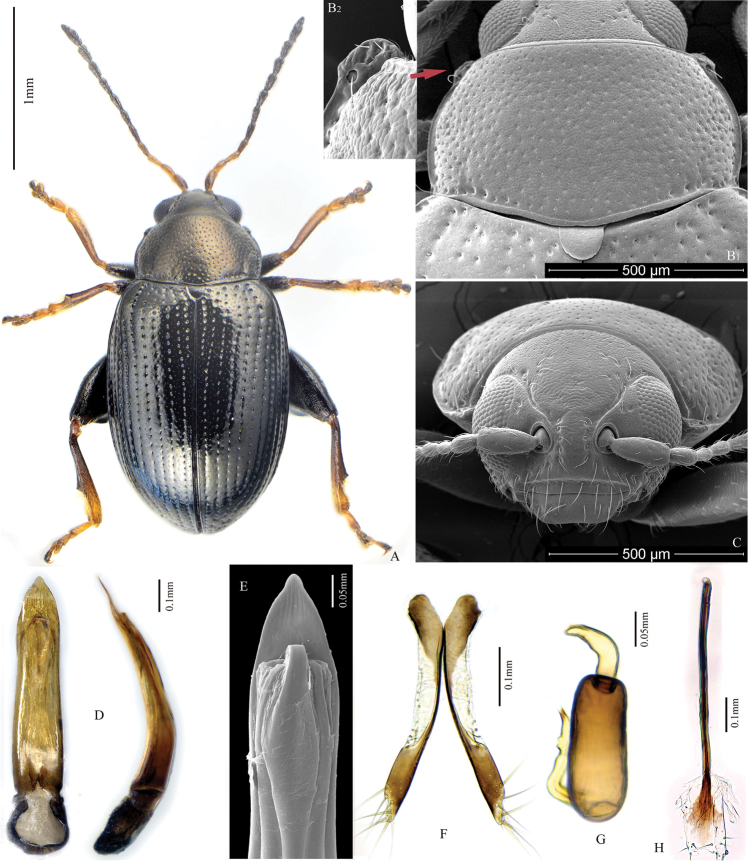
*Chaetocnema salixis*. **A** Male habitus **B1** Prothorax, dorsal view **B2** frontolateral angle of pronotum **C** Head, frontal view **D** Adeagus, ventral and lateral view **E** Apical part of Adeagus, dorsal view **F** Vaginal palpus **G** Spermatheca **H** Tignum.

Base of pronotum with two obscure longitudinal impressions visible only near basal margin. Deep row of large punctures at base of pronotum present on sides, lacking in middle. Pronotal base slightly expanded in middle. Lateral sides of pronotum slightly convex with maximum width near base. Anterolateral prothoracic callosity protruding laterally, forming a sharp angle laterally (Fig. 3: B2). Posterolateral prothoracic callosity poorly developed. Diameter of pronotal punctures 2 to 4 times smaller than distance between them.

Elytra with convex sides. All rows of punctures regular. Elytral humeral callus well developed. Interspace between stria of punctures smooth and glabrous. Numbers of stria of minute punctures on each interspace: 2.

First male protarsomere slightly larger than second one. First male protarsomere, length to width ratio: 1.74–1.78. First and second male protarsomeres, length to length ratio: 1.60–1.80. First and second male protarsomeres, width to width ratio: 1.04–1.20. First male protarsomere, width at apex to width at base: 1.50–1.53. Length of metatibia to distance between denticle and metatibial apex: 2.28–2.36. Large lateral denticle on metatibia sharp. Metatibial serration proximal to large lateral denticle absent or obscure. First male metatarsomere, length to width ratio: 2.80–3.40. First and second male metatarsomeres, length to length ratio: 2.03–2.15. First and second male metatarsomeres, width to width ratio: 0.87–0.97. Third and fourth male metatarsomeres, length to length ratio: 0.52–0.55.

Apical part of median lobe in ventral view narrowing gradually with polygonal line on sides. Ventral longitudinal groove absent. Apical denticle in ventral view absent. Minute transverse wrinkles on ventral side absent. Median lobe in lateral view evenly and strongly curved, slightly sinusoidal near apex. Maximal curvature of median lobe in lateral view situated medially.

Spermathecal receptacle pear-shaped. Basal part of spermatheca duct straight. Spermathecal pump much shorter than receptacle. Apex of spermathecal pump cylindrical or pointed. Spermathecal pump attached to middle of receptacle top. Basal part of receptacle about as wide as apical. Posterior sclerotization of tignum spoon-shaped, wider than mid section. Mid section of tignum nearly straight. Anterior sclerotization of tignum about as wide as mid section. Apex of vaginal palpus subdeltoid, with lateral side slightly arching. Sides of mid part of vaginal palpus (before apex) narrowing from base, slightly widening towards apex. Anterior sclerotization of vaginal palpus nearly parallel. Anterior end of anterior sclerotization broadly rounded. Length of posterior sclerotization greater than width. Width of posterior sclerotization smaller than width of anterior sclerotization.

##### Type material.

Holotype: 1♂, Yangxian, Qinling Mountains, **Shannxi**, alt.1700m, VII.2013, Leg.Yongying Ruan, host: *Salix* sp. "*Chaetocnema salixis* sp. n., Des. Ruan, Konstantinov, Yang. 2014" (IZCAS).

Paratypes (all in IZCAS, except those that are indicated as USNM): 2♂4♀, Qinling Mountain, **Shannxi**, VII–VIII.2013, Leg. Yongying Ruan, host: *Salix* sp.; 3♂ 4♀ same label except Leg. A. Konstantinov (USNM); 1♂2♀, Shatang forestry station, Zhouqu, **Gansu**, alt.2400m, 5–27.VII.1998, Leg. Shuyong Wang et al.; 1♀, Bifenggou, Bikou, Wenxian, **Gansu**, 25.VII.1998, alt.2360m, Leg. Xingke Yang; 1♀, Daheba, Tanchang, **Gansu**, 5.VII.1998, alt.1700–2350m, Leg. Shuyong Wang; 4♀, Miyaluo, Lixian, **Sichuang**, alt.2780–3300m, 7.VII.1963, Leg. Xuezhong Zhang; 19♂48♀, Sanshenggou, Wolong, **Sichuang**, alt.2500–2700m, 6–8.VIII.1983, Leg. Shuyong Wang.

#### Chaetocnema yulongensis

Taxon classificationAnimaliaColeopteraChrysomelidae

Ruan, Konstantinov & Yang sp. n.

http://zoobank.org/0C04C63E-43BA-4440-89A6-4DCA2F93237E

Figure 4

##### Etymology.

We named this species after the “Yulong snow mountain” in Yunnan province where the holotype was collected.

##### Host plants.

Unknown.

##### Distribution.

China, Yunnan.

##### Diagnosis.

*Chaetocnema yulongensis* and *Chaetocnema deqinensis* are extremely alike externally, but differ significantly in the shape of the median lobe. In *Chaetocnema yulongensis*, maximal curvature of the median lobe in lateral view is situated medially; apical end of the median lobe is narrowly rounded; the suprafrontal sulcus is obtuse in the middle. In *Chaetocnema deqinensis*, the maximal curvature of the median lobe is situated apically; the apical end of the median lobe is broadly rounded; the superfrontal sulcus is absent in the middle.

##### Description.

Body length: 1.66–2.15 mm, without head: 1.46–1.99 mm. Body width: 0.92–1.17 mm. Length of antenna to length of body: 0.60–0.61 mm. Elytron length (along suture) to width (maximum): 2.54–2.65. Pronotum width (at base) to length: 1.72–1.73. Length of elytron to length of pronotum (along middle): 3.25–3.30. Width of elytra at base (in middle of humeral calli) to width of pronotum at base: 1.10–1.15. Maximum width of elytra to maximum width of pronotum: 1.41–1.43.

Color of elytron copperish, similar to color of pronotum and head. Antennomere 1 partly dark brown. Antennomeres 2–4 completely yellow. Antennomere 5 partly brown. The remaining antennomeres brown. Protibia partly brown. Meso- and metatibia partly brown. Femora brown.

Head hypognathous. Frontal ridge between antennal sockets narrow and convex. Frontolateral sulcus present. Orbital sulcus deep. Suprafrontal sulcus deep laterally, shallow in middle. Suprafrontal sulcus obcordate. Ratio of width of frontal ridge (excluding margin) to width of antennal socket (excluding margin): 0.86–0.90. Width of orbital sulcus to width of frontolateral sulcus: 0.54–0.61. Numbers of punctures on vertex: 8–12. Numbers of punctures on orbit: 2. Numbers of hairs along frontolateral sulcus: 8–9. Numbers of hairs on frons (triangle area surrounded by frontolateral sulcus and clypeus): 0. Numbers of hairs on clypeus: 7. Numbers of hairs on labrum: 6. Anterior margin of labrum slightly concave in middle.

Base of pronotum with two obscure longitudinal impressions visible only near basal margin. Deep row of large punctures at base of pronotum present on sides, lacking in middle. Pronotal base evenly convex. Lateral sides of pronotum slightly convex with maximum width near base. Anterolateral prothoracic callosity protruding laterally, forming a round angle fronto-laterally. Posterolateral prothoracic callosity poorly developed. Diameter of pronotal punctures 2 to 4 times smaller than distance between them.

Elytra with convex sides. All rows of punctures regular and single including scutellar row. Elytral humeral callus well developed. Interspaces between stria of punctures smooth and glabrous. Numbers of lines of minute punctures on each interspace: 2.

First male protarsomere slightly larger than second one. First male protarsomere, length to width ratio: 1.64–1.70. First and second male protarsomeres, length to length ratio: 1.57–1.62. First and second male protarsomeres, width to width ratio: 1.03–1.04. First male protarsomere, width at apex to width at base: 1.53–1.58. Length of metatibia to distance between denticle and metatibial apex: 2.38–2.41. Large lateral denticle on metatibia sharp. Metatibial serration proximal to large lateral denticle present, obtuse. First male metatarsomere, length to width ratio: 2.63–2.73. First and second male metatarsomeres, length to length ratio: 1.91–2.04. First and second male metatarsomeres, width to width ratio: 0.93–0.96. Third and fourth male metatarsomeres, length to length ratio: 0.60–0.61.

Apical third of median lobe narrowing with polygonal line on sides. Apical part in ventral view narrowing abruptly. Ventral longitudinal groove absent. Apical denticle in ventral view absent. Minute transverse wrinkles of ventral side absent. Median lobe in lateral view evenly and slightly curved with apex sinuated. Maximal curvature in lateral view situated medially.

Spermathecal receptacle pear-shaped. Basal part of spermathecal duct straight. Spermathecal pump much shorter than receptacle. Apex of spermathecal pump cylindrical. Spermathecal pump attached to middle of receptacle top. Basal part of receptacle wider than apical. Posterior sclerotization of tignum spoon-shaped, wider than mid section. Mid section of tignum nearly straight. Anterior sclerotization of tignum narrower than mid section. Apex of vaginal palpus subdeltoid, with lateral sides slightly arching. Sides of mid part of vaginal palpus (before apex) narrowing from base, slightly widening towards apex. Anterior sclerotization of vaginal palpus narrowed at apex and base, widened at middle. Anterior end of anterior sclerotization narrowly rounded. Width of posterior sclerotization smaller than width of anterior sclerotization.

**Figure 4.**
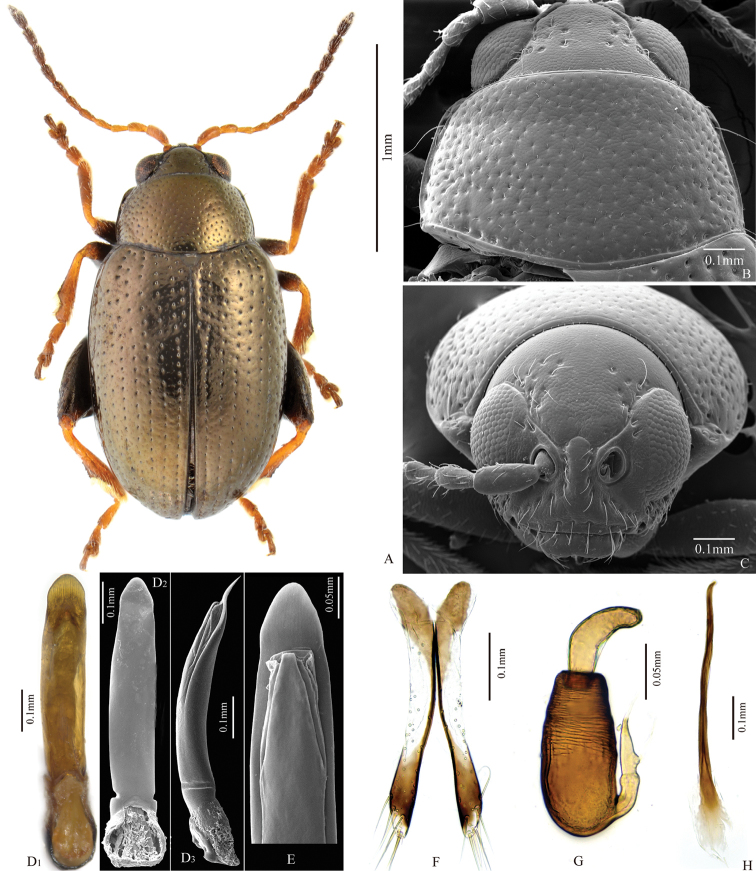
*Chaetocnema yulongensis*. **A** Male habitus **B** Prothorax, dorsal view **C** Head, frontal view **D1–D2** Adeagus, ventral view **D3** Adeagus, lateral view **E** Apical part of Adeagus, dorsal view **F** Vaginal palpus **G** Spermatheca **H** Tignum.

##### Type material.

Holotype: 1♂, 1) China: Yulong Mountain, Lijiang, **Yunnan**, alt.2700m. 2) 1984.VII.21, Leg.Shuyong Wang; 3) Holotype, *Chaetocnema yulongensis* sp. n., Des. Ruan, Konstantinov, Yang. 2014. (IZCAS)

Paratypes (all in IZCAS, except those that are indicated as USNM or SYSU): 8♂10♀, China: Yulong Mountain, Lijiang, **Yunnan**, alt.2700m, 1984.VII.21, Leg.Shuyong Wang, 1♀ USNM; 2♂5♀, Gezha, Zhongdian, **Yunnan**, alt.3150m, 1981.VIII.4, Leg.Shuyong Wang, 1♂ USNM; 1♂5♀, Xiaozhongdian, **Yunnan**, alt.2500–3200m, 1984.VIII.5, Leg.Shuyong Wang; 4♀, Lidiping,Weixi, **Yunnan**, alt.3400m, 1984.VIII.13, Leg.Shuyong Wang, 1♀ USNM); 1♂, Fengyi, **Yunnan**, alt.2000m, 1955.VI.1, Leg.B. Popov; 2♂, Xiaguan, **Yunnan**, alt.2050m, 1955.V.30, Leg.B. Popov; 6♂, Kunming, **Yunnan**, alt.1900m, 1940.VII.4, Leg.J. L. Gressitt (SYSU).

#### Chaetocnema deqinensis

Taxon classificationAnimaliaColeopteraChrysomelidae

Ruan, Konstantinov & Yang sp. n.

http://zoobank.org/A17ADFB8-C1C3-4E3B-8A51-A3307B8F1A83

Figure 5

##### Etymology.

This species is named after the type locality situated in Deqin county of Yunnan province.

##### Host plants.

*Duchesnea indica* (Andr.) Focke (Rosaceae).

##### Distribution.

Guizhou, Yunnan.

##### Diagnosis.

*Chaetocnema deqinensis* resembles *Chaetocnema yulongensis*. It can be separated from the latter by the following characters: maximal curvature of median lobe in lateral view situated close to apex; apical end of median lobe broadly rounded; suprafrontal sulcus absent in middle. In *Chaetocnema yulongensis*, maximal curvature of median lobe in lateral view situated medially; apical end of median lobe narrowly rounded; suparfrontal sulcus obtuse in middle.

##### Description.

Body length: 1.80–2.15 mm, without head: 1.60–1.95 mm. Body width: 0.90–1.10 mm. Length of antenna to length of body: 0.64–0.65 mm. Elytron length (along suture) to width (maximum): 2.40–2.60. Pronotum width (at base) to length: 1.60–1.60. Length of elytron to length of pronotum (along middle): 2.95–2.97. Width of elytra at base (in middle of humeral calli) to width of pronotum at base: 1.14–1.15. Maximum width of elytra to maximum width of pronotum: 1.46–1.48.

Color of elytron bronzish, similar to color of pronotum and head. Antennomere 1 partly dark brown. Antennomeres 2–4 completely yellow. Antennomere 5 partly brown. The remaining antennomeres brown. Tibia partly brown. Profemora and mesofemora light brown. Metafemora brown.

Head hypognathous. Frontal ridge between antennal sockets narrow and convex. Frontolateral sulcus present. Suprafrontal sulcus deep laterally, absent in middle. Orbital sulcus deep. Ratio of width of frontal ridge (excluding margin) to width of antennal socket (excluding margin): 1.04–1.07. Width of orbital sulcus to width of frontolateral sulcus: 0.88–0.90. Numbers of punctures on vertex: 8–10. Numbers of punctures on orbit: 4. Numbers of hairs along frontolateral sulcus: 9–10. Numbers of hairs on front (triangle area surrounded by frontolateral sulcus and clypeus): 0. Numbers of hairs on clypeus: 9. Numbers of hairs on labrum: 6. Anterior margin of labrum slightly concave in middle.

Base of pronotum with two obscure longitudinal impressions visible only near basal margin. Deep row of large punctures at base of pronotum present on sides, lacking in middle. Pronotal base evenly convex. Lateral sides of pronotum slightly convex with maximum width near base. Anterolateral prothoracic callosity protruding laterally, forming a round angle frontolaterally. Posterolateral prothoracic callosity poorly developed. Diameter of pronotal punctures 2 to 4 times smaller than distance between them.

Elytra with convex sides. All rows of punctures on elytron regular and single. Interspaces between stria of punctures smooth and glabrous. Numbers of lines of minute punctures on each interspace: 2. Elytral humeral callus well developed.

First male protarsomere slightly larger than second. First male protarsomere, length to width ratio: 1.80–1.86. First and second male protarsomeres, length to length ratio: 1.51–1.57. First and second male protarsomeres, width to width ratio: 0.90–0.92. First male protarsomere, width at apex to width at base: 1.60–1.90. Length of metatibia to distance between denticle and metatibial apex: 2.30–2.80. Large lateral denticle on metatibia sharp. Metatibial serration proximal to large lateral denticle present, obtuse. First male metatarsomere, length to width ratio: 2.68. First and second male metatarsomeres, length to length ratio: 1.60–1.70. First and second male metatarsomeres, width to width ratio: 0.83–0.85. Third and fourth male metatarsomeres, length to length ratio: 1.45–1.60.

Apical part of median lobe in ventral view narrowing abruptly with obscure polygonal line on sides. Ventral surface of apical fourth of median lobe deeply concave. Ventral longitudinal groove absent. Apical denticle in ventral view absent. Minute transverse wrinkles on ventral side absent. Median lobe in lateral view unevenly curved, sinusoidal near apex. Maximum curvature in lateral view situated apically.

Spermathecal receptacle pear-shaped. Basal part of spermathecal duct straight. Spermathecal pump much shorter than receptacle. Apex of spermathecal pump cylindrical. Spermathecal pump attached to middle of receptacle top. Basal part of receptacle wider than apical. Posterior sclerotization of tignum spoon-shaped, wider than mid section. Mid section of tignum nearly straight or slightly curved. Anterior sclerotization of tignum about as wide as mid section. Apex of vaginal palpus subdeltoid, with lateral side slightly arching. Sides of mid part of vaginal palpus (before apex) narrowing from base, slightly widening towards apex. Anterior sclerotization of vaginal palpus nearly parallel. Anterior end of anterior sclerotization narrowly rounded. Length of posterior sclerotization greater than width. Width of posterior sclerotization greater than width of anterior sclerotization.

**Figure 5.**
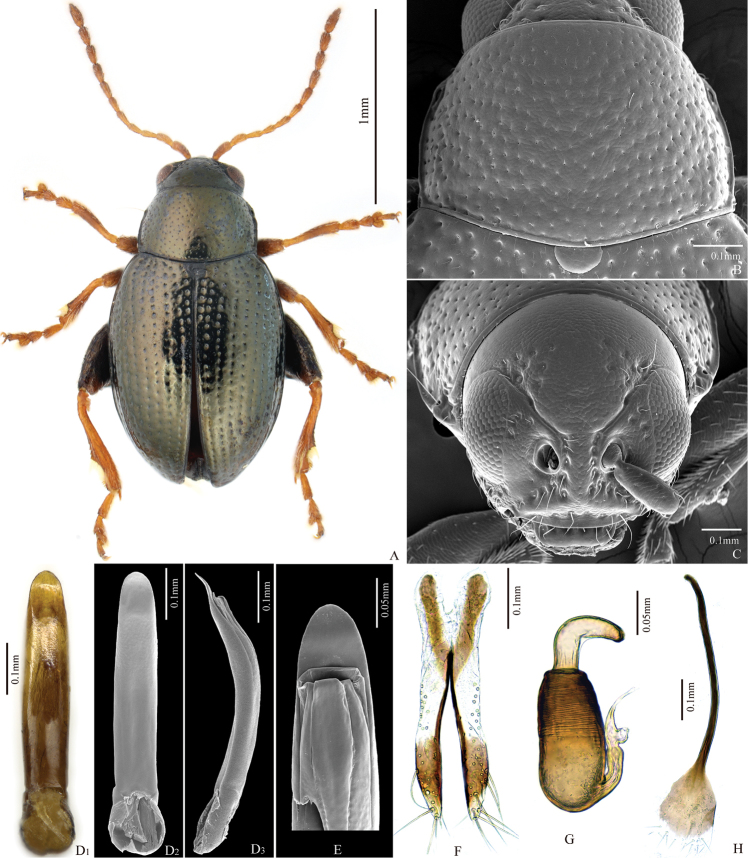
*Chaetocnema deqinensis*. **A** Male habitus **B** Prothorax, dorsal view **C** Head, frontal view **D1–D2** Adeagus, ventral view **D3** Adeagus, lateral view **E** Apical part of Adeagus, dorsal view **F** Vaginal palpus **G** Spermatheca **H** Tignum.

##### Type material.

Holotype: 1♂, China, east slope of Baiman snow mountain, Deqin, **Yunnan**, Alt.3300m, 28.VIII.1981, Leg. Shuyong Wang, host: *Duchesnea indica*. "*Chaetocnema deqinensis* sp. n., Des. Ruan et al. 2014".

Paratypes (all in IZCAS, except those that are indicated as USNM): 8♂23♀, Baiman snow mountain, Deqin, **Yunnan**, Alt.3300m, 28.VIII.1981, Leg. Shuyong Wang, host: *Duchesnea indica*, 1♀ (USNM); 1♂, Liuku, **Yunnan**, Alt.900m, 13.VI.1981, Leg. Shuyong Wang; 1♂, Zhiben Mountain, Yunlong, **Yunnan**, Alt.2250m, 21.VI.1981, Leg. Shuyong Wang; 1♂1♀, Xinzhu, Ludian, Lijiang, **Yunnan**, Alt.2800m, 29.VII.1981, Leg. Shuyong Wang; 1♀, Baoshan, **Yunnan**, Alt.1500m, 18.VI.1981, Leg. Shuyong Wang; 1♂, Heilongtang, Kunming, **Yunnan**, Alt.2000m, 14.V.1981, Leg. Shuyong Wang (USNM); 1♂4♀, Tsengyih, Meitan, **Guizhou**, 15.VII. 1940, Leg. Gressitt (SYSU).

**Figure 6.**
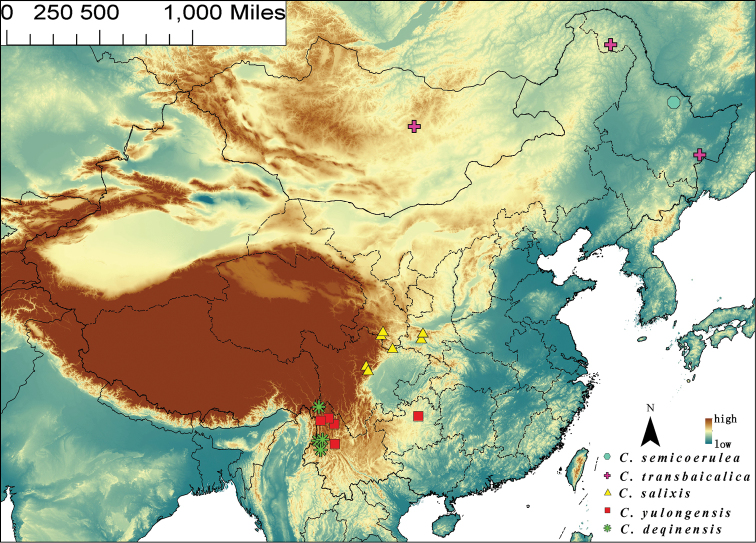
Map of mainland China, illustrating species distribution; *Chaetocnema semicoerulea* = blue octagon; *Chaetocnema transbaicalica* = purple crosses; *Chaetocnema salixis* = yellow triangles; *Chaetocnema yulongensis* = red squares; *Chaetocnema deqinensis* = green stars.

## Supplementary Material

XML Treatment for Chaetocnema semicoerulea

XML Treatment for Chaetocnema transbaicalica

XML Treatment for Chaetocnema salixis

XML Treatment for Chaetocnema yulongensis

XML Treatment for Chaetocnema deqinensis
